# Dr. Bluff

**Published:** 1839-01

**Authors:** 


### DR. BLUFF, OF AACHEN.

MATHIAS JOSEPH BLUFF, the son of poor parents, was born at Cöln, February 5, 1805. Having discovered early indications of talent, he was taken notice of by Dr. Sprögel, and placed in the way of acquiring knowledge. Having passed some years at the Gymnasium, he went to the university of Bonn in 1822, where he remained three years, studying medicine, and devoting his spare hours to the study of botany, of which he had been long fond. An early proof of his devotion to this study was the publication, in 1825, together with his friend Dr. Fingerhuth, of the first part of the "Compendium Floræ Germanicæ," since completed by Nees von Esenbeck and Schauer. From Bonn, Bluff went to Berlin, where he took his degree of M.D. in December, 1826; his thesis being "De absorptione Cutis." He first commenced practice in the small town of Gangelt, in 1827; but exchanged it for the neighbouring town of Geilenkirchen, in 1829. He remained here till 1832, when he again removed to Aachen, where he enjoyed great reputation and good practice until his death, which took place on the 5th June, 1837, in consequence of typhus fever. Dr. Bluff was a man of great taste, learning, and industry. Besides numerous communications in various medical journals, he was author of the following works:

1. Entwicklungs-Combinationen organischer Wesen. Cöln, 1827.—2. Pastoralmedizin. Aachen, 1827.—3. Ueber die Heilkrafte der Küchengewächse. Nürnberg, 1828.—4. Ueber die Krankheiten als Krankheits-ursachen. Aachen, 1829.—5. Synonymia Medicaminum. Lipsiæ, 1831.—6. Esquirol's Mordmonomanie (Transl.) mit Zusatzen. 1831.—7. Helkologie. Berlin, 1832.—8. Velpeau, die Convulsionen (Transl.) mit Zusatzen. Aachen, 1835.—9. Leistungen und Fortschritte der Medicin in Deutschland. 4 vols. 1832-6.—10. Reform der Medicin. 2 vols. Leipzig, 1836, 1837.

---

